# Plantation of Teeth

**Published:** 1897-08

**Authors:** Louis Jack


ARTICLE III.

### PLANTATION OF TEETH.

---

BY DR. LOUIS JACK, BEFORE THE ACADEMY OF STOMATOLOGY.

---

The planting of teeth is devisable into three classes:

1. *Transplantation*, where teeth removed from one subject are with proper precautions placed in the alveolus of another immediately after the extraction of a diseased or fractured tooth.

2. *Replantation*, where any given tooth of a person for various reasons may have been removed, and, after certain manipulation of the tooth and treatment of the socket, replaced.

3. *Implantation*, where an artificial alveolus is made by trephining the process at a vacant place, in which opening a suitable natural tooth is introduced.

*Transplantation* has been credited to Dr. John Hunter, but undoubtedly had been done before him. Directly after his demonstration of the feasibility of the procedure the operation was frequently performed in England, France, and to some extent in this country. The required conditions at that time were that the tooth to be transplanted should in size and form be as near as attainable the same as the one to be substituted, and necessitated one person, for a consideration, to yield the organ for the benefit of another. The removal of the tooth and the substitution were made immediately, it being considered essential that the operation be done under conditions favoring direct union.

This operation soon fell into disuse for reasons which are now clear. Dangerous inflammation frequently occurred by the transmission of disease from one subject to another, and more frequently, probably by introduction of pathogenic germs, serious disorders, sometimes followed by death, ensued.

Apparently the first described attempts in this country of the transplantation of dried teeth which had been long removed appeared in a paper, by Dr. S. P. Cutler, read before the Odontological Society of New York, January 16th, 1877. An abstract of cases is that two waste upper bicuspids were inserted side by side. After six months, on account of pain in the maxilla, the second bicuspid was removed, requiring considerable force to displace it. The first was permitted to remain.

Case II.—A first bicuspid was removed; the roots broke off, were then drilled out, and the septum of bone between them drilled away. An old tooth was fixed in the socket, which, after ten months, was firm.

Here, in passing, occurs not only the earliest attempt to use dried teeth, but also, by drilling the socket to change the form, the first suggestion of implantation appears.

For the report and discussion, see *Dental Cosmos*, vol. xix, p. 258 *et seq*.

It should be noted here that John Hunter stated that many dentists prefer dead teeth, and, further, that he had seen dead teeth become perfectly firm after insertion, and do service for many years.

*Replantation* is not traceable to any authority for its beginning. It probably had its rise in the natural tendency to replace teeth dislocated by accident or removed by mistake. Our literature shows that for this purpose it was generally successful without the care which would now be exercised to insure a safe result. Within recent periods it has been performed as a means of effecting the the cure of chronic alveolar abscess and other disorders of the root. The results have generally been reported as curative. This practice has been confined to the more careful and judicious practitioners, which is borne out by an examination of the literature of replantation.

Successful treatment in this manner and for this latter purpose became coincident with the scientific practice of aseptic surgery. Without careful consideration of the application of the principles governing asepsis, replantation in disordered conditions of the teeth and peridental membrane would be as liable to failure and as dangerour as transplantation in the previous century.

The *rationale* of the treatment here would appear to be, if we exclude the influence of shortening the root and occluding the root-canal, that the traumatism induces for a time an acute or subacute condition for the previous chronic one.

The relation of failures in replantation and transplantation previous to 1885 should be ignored. A tooth extracted and handled necessarily becomes an infected thing, and without disinfection its presence in the tissues of the alveolus involves a continuous struggle between the vital forces of the part and the development of bacteria. But through all these influences occasionally replanted and transplanted teeth remained for many years. I know of a man who had an inflamed lateral incisor removed. The pulp had been devitalized. Deploring the loss, he replaced it on retiring for the night. On arising it had escaped. Not to be outdone, he tore from his handkerchief a narrow part of the selvage, and with this tied the tooth to one of the adjacent ones. It remained for fourteen years.

*Implantation* was undoubtedly originated by Dr. Younger, whose claim has not been publicly disputed. The date of his first operation was June 15th, 1885. The general procedures of this operation as practiced by him are so familiar that it is needless here to repeat them.

The result of the fixation of an implanted tooth when it has been properly adjusted and held in position has become indisputable. The evidence presented by men of reliability and sincerity of purpose has too much accumulated to fail to be convincing as to the feasibility of implantation.

There are certain requirements already established which govern success. The artificial opening must have the depth to give correct occlusion, and be but little larger than the root of the tooth to be inserted. The opening in the gum should be smaller than the cross-section area at the cervix of the tooth. The mouth should be disinfected. The operator's hands and all instruments must be put in an aseptic condition. The tooth should be rendered impersonal and aseptic by being heated in a disinfectant fluid. The tooth should not have shreds of peridental membrane on it, but should not be scraped clean of the adherent portion of the membrane. During intervals of the operation the socket should be filled with a tent of the antiseptic fluid in use. When the tooth is inserted it is better that some force be employed to push it into place. It should then be securely fixed in position by some means which will give immobility.

With the exception of the formation of the opening in the gum and the trephining of the process, implantation has no essential differentation from the requirements governing transplantation and replantation.

With this outline I will proceed to describe the methods I have pursued in the cases under my care.

The most important qualification connected with these operations is the means to secure thoroughness of asepsis. The usual reliance has been on bichloride of mercury. I have avoided its use on account of its irritating influence and its depression of the vital force of the tissues, and have relied on the aqueous solution of hydronapthol and the aqueous solution of acetanilid.

The former is souluble in the proportions of 1 to 300.

The latter is freely soluble in the ratio of 1 to 200. To effect the solution of hydronaphthol it is first dissolved in alcohol, when it becomes entirely miscible in water. In these proportions neither is irritating to the tissues, and, when heated, thoroughly effect the disinfection of the tooth, the hands, and instruments. The importance of heating disinfectant solutions to increase their power has been well established. (For example, with bichloride of mercury 1 to 20,000 of urine at 104 degrees F. prevents fermentation. Anthrax spores lose their infecting power in weak solutions of the ordinary antiseptics at 170 degrees F.) I place the tooth in either of the above disinfectant solutions and raise the temperature to the boiling-point. The instruments likewise are heated in the same manner. These and the tooth are kept in the fluid in the intervals which occur during the surgical procedures.

Generally I have subjected the selected tooth to this process for a short time after opening the pulp-chamber to disinfect the root-canals.

The importance of securing immobility of the planted tooth cannot be overestimated, since the structural changes which produce the union of the tooth cannot be expected without a state of rest. It must be as essential here as in fractures, for the process of ankylosis must be analogous to the process of the union of bones. Therefore, I emphasize the necessity for the absolute fixation. I take an impression in plaster, and on the cast make a study of the case. The tooth is fitted in the plaster to correct relations with adjoining and occluding teeth. It is then secured in place, when a metal cast is taken, and on this is fitted a gold splint of whatever form and with whatever attachments may give stability. This is secured by clasps or by wire ligatures to the other teeth at the cervix. Additional security and cleanliness is effected by setting it in place with oxiphosphate.

For the front teeth, which are the most difficult to fix, I open into the pulp chamber on the lingual surface and insert a wire parallel with this face. The splint is formed to pass between the pin and the tooth. This, if the splint is secured to the adjacent teeth, establishes the fixation in each direction.

These mechanical preparations may appear to some to be unnecessarily tedious. In a question where success depends on careful observances these precautions are essential.

To protect the surface of the root from injury during these procedures, it is covered with a strip of mucilage paper wrapped on it, or a layer of floss silk. To prevent drying out, the cast is covered with damp paper or kept in a box among damp paper.

The final operation on the mouth is simple, and is speedily done. It is preceded by every aseptic precaution.

In respect of replantation and transplantation, I invariably have broken up the remains in the alveolus of the peridental membrane. My impressions have been that this places these cases in the same relations which occur in implantation. The grounds for this procedure are that the experience of those who have replanted and transplanted teeth were of very frequent failure by early resorption, while the implantations when carefully performed have given favorable results.

I have implanted four cases; of these, three became fixed with apparent calcific support.

No. 1. Right upper central, male; operation April 20th, 1889. In a few months it became firm and remained so for six years and five months, when it suddenly became loose and fell away. At the time of the operation the patient was in good health, but for a year or more before the resorption he had failed in vigor.

No. 2. Right upper lateral, male; operation January 12, 1895. The tooth became attached quickly, and is now resonant.

No. 3. Right upper central, same patient as No. 2; operation same date. On account of the occlusion of the lower tooth on the tongue of the splint, secure attachment failed. It was removed December 7th, 1895 when another tooth was inserted. It is notable that the alveolus had contracted as the root became diminished by resorption and required deepening and enlargement to receive the new tooth. The location of these teeth had been edentulous for thirty years.

No. 4. Right upper bicuspid, male; operation April 22d, 1895. Progressed favorably; splint removed in four months. Tooth now very resonant.

My transplantations have been five.

No. 1. Left upper lateral, female; operation October 22d, 1892. Tooth dead; deep pocket on palatal aspect; fistula on gum on labial aspect; apex of root much denuded of membrane and quite loose. Deepened the alveolus and firmly splinted the case; splint removed in five months; calcification was not established till four months afterward. The tooth remains perfectly firm, and is very resonant.

No. 2. Right lower lateral; operation October 10th, 1894. Profound pyorrhea, with much resorption of margins of alveolus; patient not of good health; tooth became fastened by connective tissue, but it failed to become firm and was removed in six months. Here I was unable to attach a splint, and relied on wire ligatures, which were not sufficient to prevent motion.

No. 3. Left upper lateral, male; operation January 12th, 1895. The root here was deeply split, the parts much separated, thus enlarging the alveolus. The gum quickly embraced the root. In a few weeks the tooth became firm; it is now very resonant. Here the face became swollen, with perceptible heat, which quickly responded to treatment.

No. 4. Left upper cuspid, female; operation March 12th 1895. Here the tooth had been nearly disconnected by pericementitis; excessive flow of pus; outer plate of alveolus very much destroyed. The alveolus was fully sprayed with the solution of acetanilid; a tent also of lint saturated with an alcoholic solution of the same was applied for a few minutes. The next day swelling of the tissue about the tooth occurred. The face over the canine fossa was suffused and swollen; refrigerated face and sprayed gum with acetanilid one-half per cent. In two days these appearances subsided; no trace of pus could be elicited by pressure over the tooth. November 9th, after eight months, the tooth being resonant, the splint was removed. This case is remarkable from the extent of resorption of the alveolus, and from the free suppuration consequent on the depth of the pockets.

No. 5. Right upper second bicuspid, male. The tooth very loose from pericementitis; process much resorbed, but no discharge of pus at the time; tooth removed November 2d, 1894; operation twenty days later. The fixation by splint was not secure from disturbance by the occlusion of the lower tooth. There is now no indication of calcification.

Of replantation I have had three cases.

No. 1. Right upper first bicuspid, female; operation November 3d, 1894. Displacement outward by pericementitis, with resorption of outer wall of alveolus. Reformed the socket to secure alignment. In three hours replaced the tooth. Removed splint in six months. The tooth is now firm and very resonant.

No. 2. Right upper lateral, female; operation January 19th, 1895. Tooth disturbed outward and posteriorly, displacing the lip, caused by pericementitis. Deepened the alveolus and reformed it to secure alignment. Management same as in previous case. The gum became slightly inflamed, which passed away in six days, and the splint was removed at end of June.

No. 3. Right upper central, female; a case of pyorrhea with enlargement of the alveolus; operation March 23d, 1885. Here, because the remaining adjacent teeth were not firm, a state of immobility could not be secured. The ultimate attachment is doubtful.

The apparent promising results in teeth deeply affected by the consequences of pericementitis is an important conclusion to be drawn from several of the above cases.

I have alluded to the means taken to keep the root clean and protect the surface from injury. If a protected root apparently free of portions of the peridental membrane be immersed in warm water or a disinfecting solution the surface slightly swells, becomes velvety to the touch, and is viscid. It is a rational conclusion that the glasma effused into the space surrounding the planted tooth must necessarily come into more intimate connection with the tooth in this condition than would be if the root were polished. Moreover, a tooth in the condition stated when removed during the operation for adjustment exhibits a considerable degree of resistance to displacement, which I have attributed largely to this condition.—*Welch's Monthly*.

